# The tumor‐to‐liver ratio of the standardized uptake value is a useful FDG‐PET/CT parameter for predicting malignant intraductal papillary mucinous neoplasm of the pancreas

**DOI:** 10.1002/ags3.12562

**Published:** 2022-03-23

**Authors:** Takeshi Utsunomiya, Kohei Ogawa, Naotake Funamizu, Katsunori Sakamoto, Jota Watanabe, Hiromi Otani, Naoto Kawaguchi, Masao Miyagawa, Hirotaka Iwaki, Yasutsugu Takada

**Affiliations:** ^1^ Department of Hepato‐Billiary‐Pancreatic Surgery Ehime University Hospital Ehime Japan; ^2^ 37119 Department of Gastroenterological Surgery Ehime Prefectural Central Hospital Ehime Japan; ^3^ 38050 Department of Radiology Ehime University Graduate School of Medicine Ehime Japan; ^4^ 38050 Department of Clinical Pharmacology and Therapeutics Ehime University Graduate School of Medicine Ehime Japan

**Keywords:** FDG‐PET/CT, high‐risk stigmata, IPMN, NLR, SUV

## Abstract

**Background:**

The present study aimed to investigate the efficacy of positron emission tomography with 18Fluoro‐deoxyglucose (FDG‐PET/CT) for predicting malignant intraductal papillary mucinous neoplasm (IPMN).

**Methods:**

The records of 88 patients pathologically diagnosed with IPMN after surgery at Ehime University Hospital and Ehime Prefectural Central Hospital from April 2009 to December 2020 were retrospectively reviewed. The patients’ characteristics, blood chemistry, and imaging examinations were evaluated as potential predictors of malignant IPMN. Of the PET/CT results, the maximum standardized uptake value (SUVmax) of the tumor, the tumor‐to‐blood pool ratio of the SUV (TBR), and the tumor‐to‐liver ratio of the SUV (TLR) were compared.

**Results:**

On pathology, the diagnosis was adenoma (IPMA) in 40 patients, high‐grade dysplasia (HGD) in 26 patients, and carcinoma (IPMC) in 22 patients. HGD and IPMC were defined as malignant IPMN. On multivariate analyses, TLR ≥ 1.3 and high‐risk stigmata were independent predictors of malignant IPMN (*P* = .001 and *P* = .007, respectively). When both HRS and TLR ≥ 1.3 were present, the positive predictive value for malignancy was 88.2%. Furthermore, TLR was significantly higher for patients with IPMC than with HGD (*P* = .039).

**Conclusion:**

TLR can be a useful predictor for differentiating benign from malignant IPMN and may be associated with postoperative outcomes.

## Graphical abstract

Although the FDG PET/CT maximum standardized uptake value (SUVmax) of the tumor is a useful parameter, it is not an absolute value but a relative value, and it is affected by various factors such as equipment and imaging protocols. In order to make SUVmax more universal, the ratio of SUVmax of the tumor to SUV mean of the liver right lobe (TLR) was investigated. The present study showed that the TLR can be a useful predictor for differentiating benign from malignant IPMNs and may be associated with outcomes, such as postoperative recurrence.

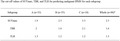

## 1 INTRODUCTION

With recent advances in computed tomography (CT) and magnetic resonance imaging (MRI), one can more easily detect pancreatic cystic lesions, and the number of incidentally identified cases is increasing.^1^, ^2^, ^3^ Intraductal papillary mucinous neoplasm (IPMN) is the most common cystic disease of the pancreas. Main duct IPMN (MD‐IPMN) is known to have high malignant potential. In a previous report, 72% of resected MD‐IPMN cases were found to show high‐grade dysplasia (HGD) or intraductal papillary mucinous carcinoma (IPMC) on pathology.^4^ In the Fukuoka guideline 2017, high‐risk stigmata (HRS) are defined as important findings suggesting malignancy in IPMN, and surgical treatment is indicated for patients with high‐risk stigmata.^5^ The recurrence rates of HGD and IPMC were reported to be 13.3% and 33.8%, respectively, whereas that of low‐grade and intermediate dysplasia was reported to be 4.0%.^6^ The size and type of invasive component, lymph node positivity, and a positive resection margin were all important risk factors for recurrence.^4^ On the other hand, surgical treatment of the pancreas has substantial morbidity and mortality. For example, the postoperative mortality rate of pancreaticoduodenectomy (PD) was reported to be 0.9%‐3.9%.^7^, ^8^, ^9^ Accordingly, it is thought that the exact preoperative diagnosis of HGD and IPMC can help determine which IPMN patients require surgery.

Positron emission tomography with 18Fluoro‐deoxyglucose (FDG‐PET)/CT has been reported to have high specificity and sensitivity in differentiating IPMA from HGD and IPMC.^10^, ^11^ Although the maximum standardized uptake value (SUVmax) of the tumor is a useful parameter, it is not an absolute value but a relative value, and it is affected by various factors such as equipment and imaging protocols.^12^ In order to reduce the effects of various factors on the SUVmax, we attempted to use the relative ratio of accumulation of the tumor to that of other regions.^13^ In this study, the relationships between preoperative findings of FDG‐PET/CT and the pathological diagnosis were investigated in an attempt to identify a more universal parameter that could be widely used in many institutions for predicting malignant IPMN.

## 2 MATERIALS AND METHODS

### 2.1 Patients

**FIGURE 1:**
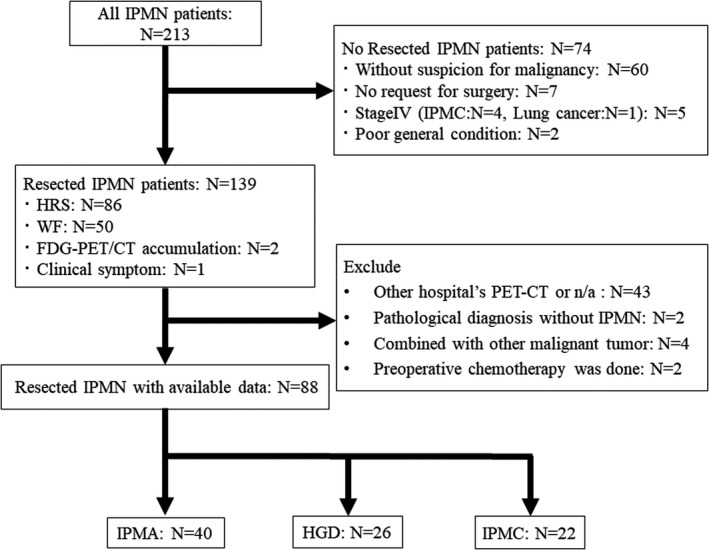
Consort diagram for case selection. From April 2009 to December 2020, 213 patients with IPMN were referred to the Department of Surgery at Ehime University Hospital and Ehime Prefectural Central Hospital. Seventy‐four patients did not undergo surgery for several reasons. A total of 139 underwent surgical resection for a preoperative diagnosis of IPMN. Fifty‐one patients were excluded from this study: 43 patients who did not undergo FDG‐PET/CT or underwent FDG‐PET/CT examination at other hospitals, 4 patients with concomitant other malignant tumors, 2 patients whose postoperative pathological diagnoses were not IPMN, and 2 patients given preoperative chemotherapy. In total, 88 patients were included in this study

From April 2009 to December 2020, 213 patients with IPMN were referred to the Department of Surgery at Ehime University Hospital and Ehime Prefectural Central Hospital. Seventy‐four patients did not undergo surgery. Reasons for not performing surgery included no malignant findings (without worrisome features or HRS) in 60 patients, no desire for surgery in seven patients, Stage IV cancers including IPMC in five patients, and poor general condition in two patients. A total of 139 patients underwent surgical resection for a preoperative diagnosis of IPMN. Fifty‐one patients were excluded from this study: 43 patients who did not undergo FDG‐PET/CT or underwent FDG‐PET/CT examination at another hospital, four patients with concomitant other malignant tumors, two patients whose postoperative pathological diagnoses were not IPMN, and two patients with preoperative chemotherapy. In total, 88 patients were included in this study (Figure 1). Data were obtained from prospectively maintained hospital databases. The databases captured all patients who underwent surgical resection for a preoperative diagnosis of IPMN. Surgical resection was indicated for IPMN with suspected malignancy or symptomatic IPMN, such as pancreatitis. As a rule, in the cases of HRS, surgery was suggested to the patient in all cases. In the cases of worrisome features, surgery was performed if a contrast‐enhanced mural nodule was evident, or if there was an enlargement or new appearance of the cyst or enhanced mural nodule. Only two patients were operated on based on high FDG‐PET/CT accumulation without HRS or worrisome features. The diagnoses of these two patients were IPMC on postoperative pathology. In this study, HGD and IPMC were defined as malignant according to the Fukuoka guideline 2017.

### 2.2 Subgroups

The study population was divided into three subgroups according to the institutions and FDG‐PET/CT equipment: 33 patients until March 2020 at Ehime University (group A), 35 patients until December 2018 at Ehime Prefectural Central Hospital (group B), and 18 patients from January 2019 to December 2020 at Ehime Prefectural Hospital (group C).

There were only two patients who were tested by new PET/CT equipment at Ehime University Hospital from April 2020 to December 2020, and they were excluded from the subgroups.

### 2.3 Protocol

#### 2.3.1 Laboratory data

Blood test results examined included peripheral blood cell fraction, albumin, C‐reactive protein, and tumor markers. Regarding the peripheral blood cell fraction and C‐reactive protein, the most improved preoperative inflammatory findings were used.

#### 2.3.2 Preoperative imaging studies

Endoscopic ultrasound (EUS), CT, MRI, and FDG‐PET/CT were performed to detect tumor lesions. IPMNs were classified as main duct (MD)‐IPMNs and branched duct (BD)‐IPMNs based on preoperative findings. Mixed type (MT)‐IPMNs were classified as MD or BD according to the more predominant component. The definitions of worrisome features and high‐risk stigmata were according to the Fukuoka guideline 2017.^5^

#### 2.3.3 FDG‐PET/CT findings

**FIGURE 2:**
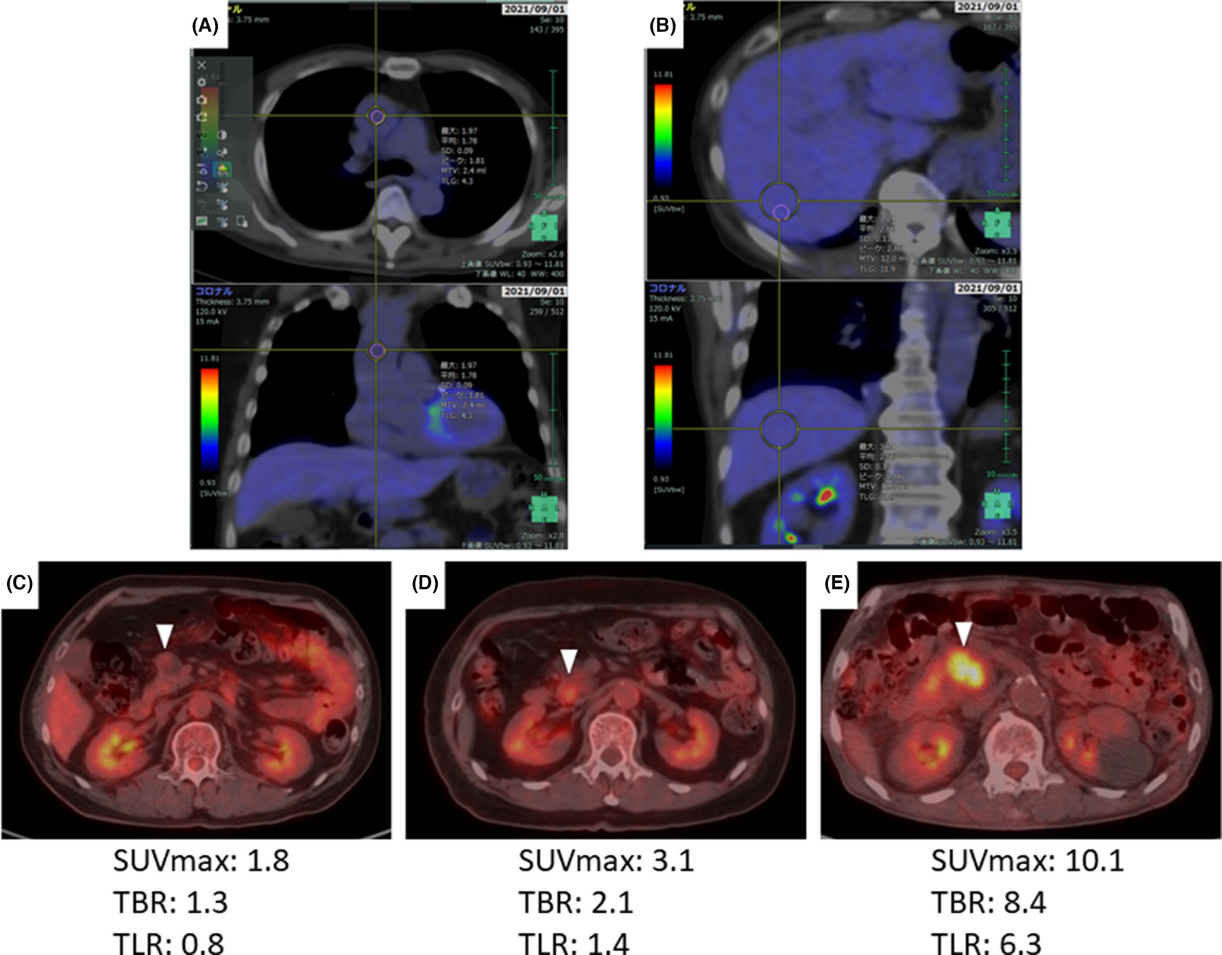
Measurements of SUV in FDG‐PET/CT. A, SUV mean measurements in the blood pool of the ascending aorta. B, SUV mean measurement in the right lobe of the liver. C, Typical example of PET‐CT image for IPMA. The white arrow indicates the site of accumulation. D, Typical example of PET‐CT image for HGD. The white arrow indicates the site of accumulation. After formalin fixation, HGD portion was widely distributed within the tumor (maximum diameter: 23 mm). The FDG‐accumulated area (maximum diameter: 14.5 mm) generally corresponded to the distribution of the HGD portion. E, Typical example of PET‐CT image for IPMC. The white arrow indicates the site of accumulation

In both institutions, basically, FDG‐PET/CT was routinely performed in all patients when considering surgery for IPMN, unless it had been already performed at other hospitals. FDG PET/CT was performed after fasting for 6 hours, with a glucose level <150 mg/dL. PET/CT imaging was obtained using a multi‐slice scanner 60‐90 minutes after intravenous administration of 3.0‐4.0 MBq of FDG per kilogram of body weight. The PET/CT equipment differed among the three subgroups: Discovery 600^®^, Discovery ST elite^®^, and Discovery IQ 2.0^®^ (GE Healthcare) for subgroups A, B, and C, respectively. The FDG dose, image acquisition, and image reconstruction were based on the protocols of the institutions and adapted to the equipment used. The SUVmax of the IPMN tumor, the tumor SUVmax to blood pool mean SUV ratio (TBR), and the tumor SUVmax to liver mean SUV ratio (TLR) were obtained.^13^ To obtain the mean SUV for the blood pool and the liver, the volumes of interest (VOIs) were drawn from the area just distal to the aortic valve in the ascending aorta and the center of the right hepatic lobe, respectively, on fusion images. Spherical regions of VOIs were approximately 2.5 cm^3^ for the blood pool and 12 cm^3^ for the liver, respectively. In addition, in patients found to have multiple IPMN lesions on PET imaging, a set of spherical VOIs was placed just around the lesion showing the highest FDG uptake. SUV mean measurements in the blood pool of the ascending aorta and in the right lobe of the liver are shown in Figure 2. Figure 2 also shows typical PET‐CT hyperintensities for IPMA, HGD, and IPMC.

### 2.4 Statistical analysis

Continuous variables are presented as medians with ranges and were compared using Mann‐Whitney *U* tests. The cut‐off values for predicting malignancy were determined by receiver operating characteristic (ROC) curve analysis and the Youden index. Significant variables on univariate analyses that correlated with malignancy on Pearson's χ^2^ test or Fisher's exact tests were included in multivariate analyses with step‐down logistic regression and likelihood tests. Regression models were calibrated using Hosmer‐Lemeshow tests. For multivariate analyses, data were screened for multicollinearity. Significance was defined as *P* <.05. All data were analyzed using SPSS^®^ version 25 (IBM, Armonk).

### 2.5 Ethical considerations

The institutional review board of Ehime University Hospital approved this study (Approval number: 1908017), which was conducted in accordance with the ethical standards established in the Declaration of Helsinki in 1995 (Brazil 2013 revision). Written, informed consent was obtained using the opt‐out principle. The nature of the study and the right of refusal to participate were disclosed to the public online. None of the authors have any conflicts of interest to disclose regarding this study.

## 3 RESULTS

For the 88 patients, the operative procedures were pancreatoduodenectomy in 55 (62.5%) patients, distal pancreatectomy in 30 (34.1%) patients, total pancreatectomy in three (3.4%) patients. Postoperative pathology showed IPMA, HGD, and IPMC in 40 (45.5%), 26 (29.5%), and 22 (25.0%) patients, respectively.

### 3.1 Comparison between the IPMA and HGD + IMPC groups

**FIGURE 3:**
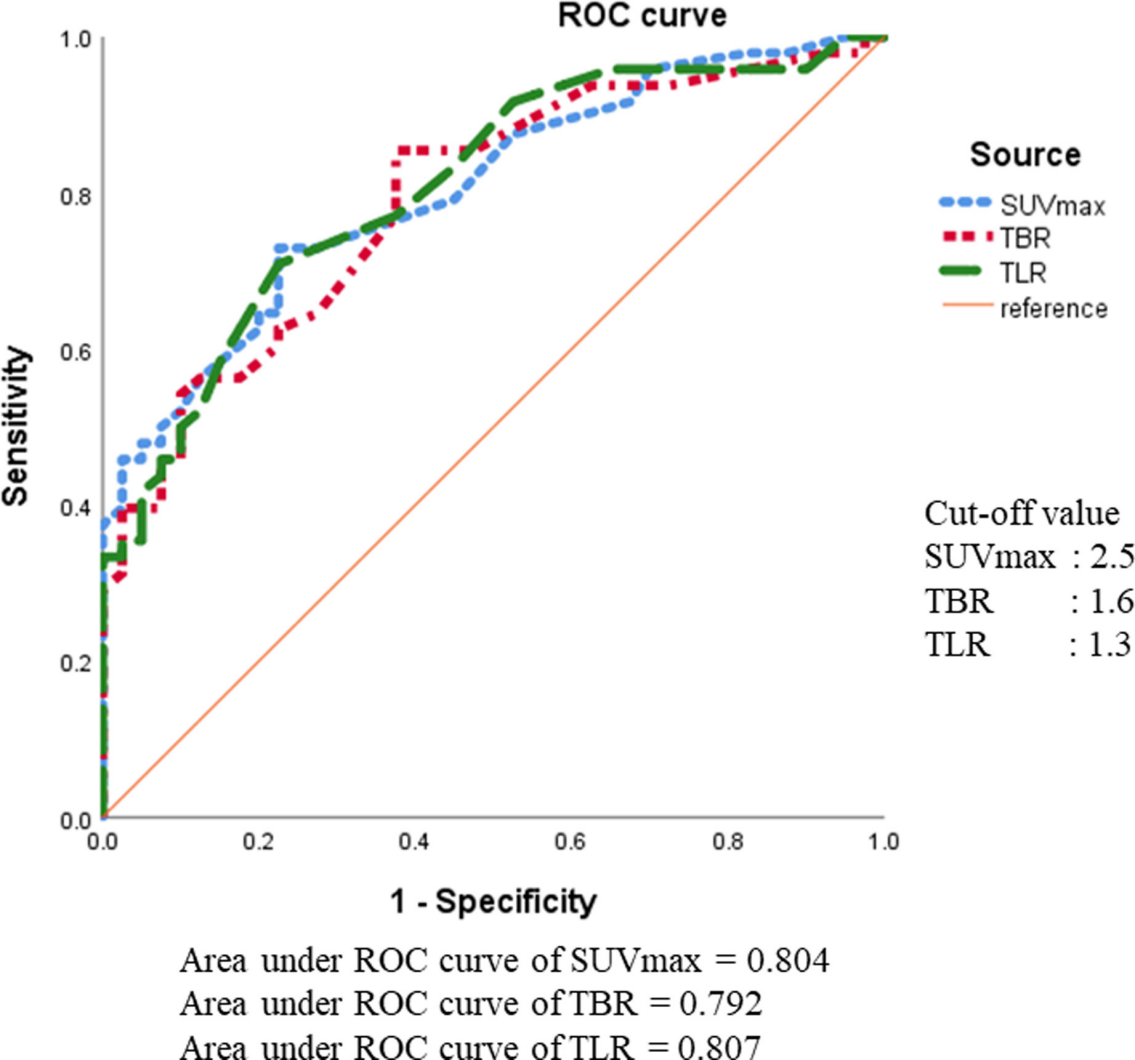
Receiver‐operating characteristic (ROC) curves of the SUVmax, TBR, and TLR for predicting HGD + IPMC cases in all 88 cases (2009‐2020). The areas under the ROC curves (AUCs) of SUVmax, TBR, and TLR are 0.804, 0.792, and 0.807, respectively. The optimal cut‐off values of SUV max, TBR, and TLR are 2.5, 1.6, and 1.3, respectively. The sensitivity and specificity are 72.9% and 77.5%, 85.4% and 62.5%, and 70.8% and 77.5%, respectively

On univariate analysis, age, sex, BMI, symptoms, neutrophil‐to‐lymphocyte ratio (NLR), CRP‐to‐albumin ratio (CAR), rates of CEA above the normal level (>5.0 ng/mL) and CA19‐9 above the normal level (>37 U/mL), and main tumor location did not differ significantly between the IPMA and HGD + IMPC groups (Table 1). The main duct type and HRS were more frequent, and mural nodule size was larger in the HGD+IPMC group (*P* =.018, *P* < .001 and *P* < .001, respectively) compared to the IPMA group. The SUVmax, TBR, and TLR were all significantly higher in the HGD + IPMC group than in the IPMA group (*P* < .001, *P* < .001, and *P* < .001, respectively). The ROC curves of SUVmax, TBR, and TLR for predicting malignancy are shown in Figure 3. The areas under the ROC curve (AUCs) were 0.804, 0.792, and 0.807 for SUVmax, TBR, and TLR, respectively. The optimal cut‐off values of SUV max, TBR, and TLR were 2.5, 1.6, and 1.3, respectively. The sensitivity and specificity were 72.9% and 77.5%, 85.4% and 62.5%, and 70.8% and 77.5%, respectively.

**TABLE 1:** Preoperative data of the IPMA group and the HGD +IPMC group

	IPMA, n = 40	HGD + IPMC, n = 48	*P* value
Age	72 (56‐88)	74.5 (48‐88)	.237
Sex, male	27 (67.5%)	36 (75.0%)	.437
BMI	23.7 (16.6‐31.2)	22.2 (16.9‐30.9)	.657
Symptomatic	7 (17.5%)	17 (35.4%)	.060
NLR	2.2 (0.6‐4.4)	2.0 (0.7‐6.6)	.688
CAR	0.015 (0.000‐0.148)	0.017 (0.000‐0.776)	.354
CEA > 5 ng/mL	3 (7.5%)	8 (17.0%), n = 47	.183
CA19‐9 > 37 U/mL	5 (12.8%), n = 39	14 (30.4%), n = 46	.052
Main location; head	27 (67.5%)	29 (60.4%)	.492
Size of mural nodule (mm)	1.0 (0‐18.0)	8.0 (0‐40.0), n = 47	<.001
Main duct type	8 (20.0%)	21 (43.8%)	.018
HRS	15 (37.5%)	38 (79.2%)	<.001
SUVmax	2.2 (1.2‐4.8)	3.3 (1.5‐14.9)	<.001
TBR	1.4 (0.7‐4.4)	2.45 (0.9‐11.9)	<.001
TLR	1.0 (0.5‐2.7)	1.7 (0.7‐8.7)	<.001

Note

Continuous variables are presented as the medians with ranges. Categorical variables are presented as the patient numbers and ratios (%).

Abbreviations: BMI, body mass index; CAR, CRP‐albumin ratio; HGD, high grade dysplasia; HRS, high risk stigmata; IPMA, intraductal papillary mucinous adenoma; IPMC, intraductal papillary mucinous carcinoma; NLR, neutrophil‐to‐lymphocyte ratio; SUVmax, maximum standardized uptake value; TBR, tumor‐to‐blood pool ratio of the SUV; TLR, tumor‐to‐liver ratio of the SUV.

**FIGURE 4:**
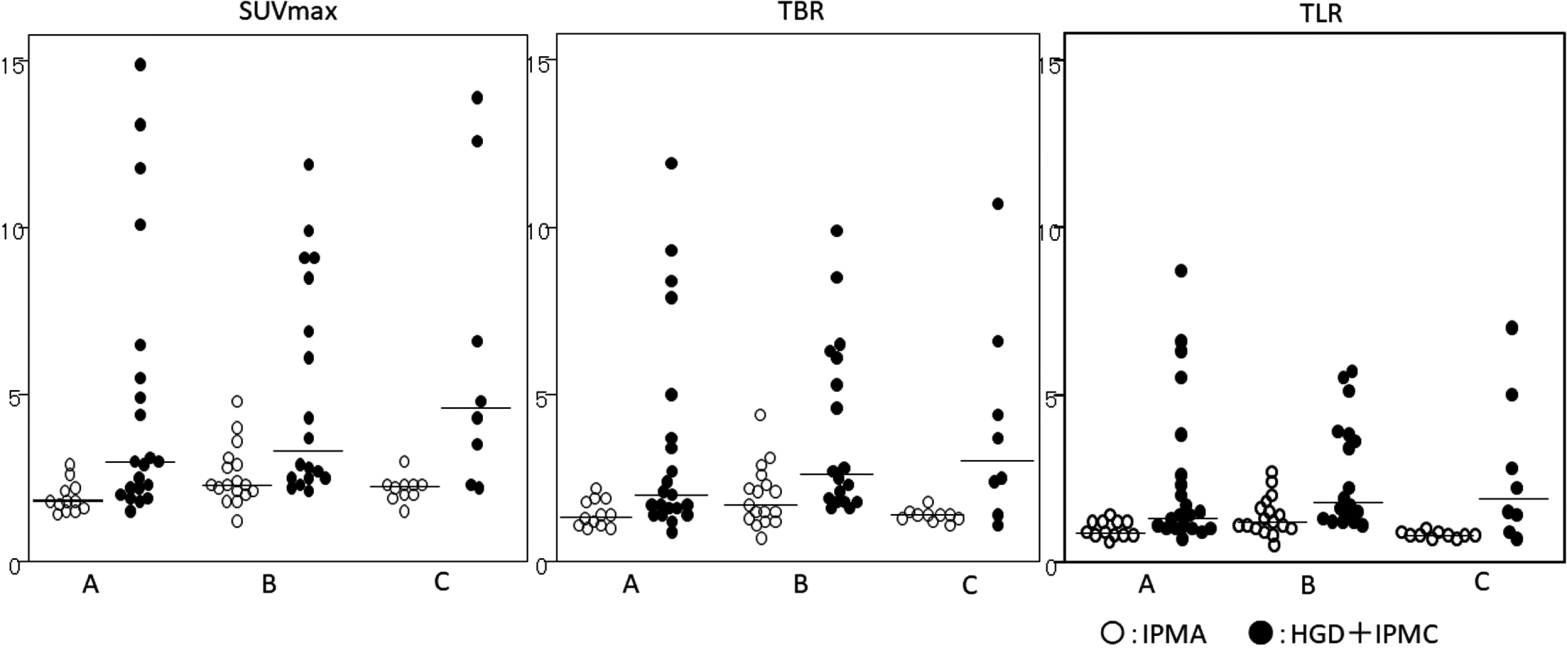
The distributions of the SUVmax, TBR, and TLR in each subgroup are shown. The lines represent the median values of each group. White circles represent IPMA, black circles represent HGD and IPMC. Subgroup A: Ehime University Hospital cases (2009‐March 2020), Subgroup B: Ehime Prefectural Central Hospital cases (2009‐2018), Subgroup C: Ehime Prefectural Central Hospital cases (2019‐2020)

Figure 4 shows the distribution of individual values of SUVmax, TBR, and TLR in each subgroup. The distribution of the SUVmax was widely different among the three subgroups, and the median values of the HGD + IPMC patients ranged from 3.0 to 4.55. The distributions of TBR and TLR were comparatively similar among the three subgroups. When the cut‐off levels for differentiation between benign and malignant IPMN were determined by ROC curve analysis separately in each subgroup (Table 2), that of SUVmax was highly variable among the three subgroups (1.9, 2.5, and 3.3). In contrast, that of TLR was almost the same (1.2 or 1.3) among the three subgroups.

**TABLE 2:** The cut‐off values of SUVmax, TBR, and TLR for predicting malignant IPMN for each subgroup

Subgroup	A (n = 33)	B (n = 35)	C (n = 18)	Whole (n = 88)^a^
SUVmax	1.9	2.5	3.3	2.5
TBR	2	1.6	2.1	1.6
TLR	1.3	1.2	1.2	1.3

Note

Subgroup A: Ehime University Hospital cases (2009‐March 2020), Subgroup B: Ehime Prefectural Central Hospital cases (2009‐2018), Subgroup C: Ehime Prefectural Central Hospital cases (2019‐2020).

Abbreviations: SUVmax, maximum standardized uptake value; TBR, tumor‐to‐blood pool ratio of the SUV; TLR, tumor‐to‐liver ratio of the SUV.

^a^ Two patients who were tested by new PET/CT equipment at Ehime University Hospital from April 2020 to December 2020 were not assigned to the subgroups.

Table 3 shows the results of multivariate analysis, which included factors found to be significant on univariate analysis. Regarding mural nodule size, the cut‐off value for predicting malignant IPMN determined by the ROC curve was 5.0 mm (sensitivity: 80.9%, specificity: 62.5%), which is consistent with the mural nodule size category of the HRS. Therefore, it was excluded from the multivariate analysis because it is a confounder of the HRS. As for parameters of PET/CT, since SUVmax, TBR, and TLR are confounding factors, and the cut‐off value of TLR was invariable among the subgroups, only TLR was included in the multivariate analysis. It was found that HRS and TLR ≥ 1.3 were the independent factors for predicting malignancy (hazard ratio: 4.106 and 5.954, 95% CI: 1.464‐11.513 and 2.146‐16.518, *P* = .007 and *P* = .001, respectively).

**TABLE 3:** Multivariate analysis for differentiation between the IPMA group and the HGD + IPMC group

	*P* value	Hazard ratio	95% CI	
Main duct type	.126	2.489	0.774	8.006
High risk stigmata	.007	4.106	1.464	11.513
TLR ≥ 1.3	.001	5.954	2.146	16.518

Abbreviation: TLR, tumor‐to‐liver ratio of the SUV.

Table 4 shows the positive predictive value for malignancy of the combination of independent predictive factors. When both HRS and TLR ≥ 1.3 were present, the positive predictive value was 88.2%. The combination of HRS and TLR ≥ 1.3 increased the positive predictive value by 16.5% compared with HRS alone.

**TABLE 4:** Positive predictive value for malignancy determined by the presence of HRS and the presence of TLR ≥ 1.3

Factor	Positive predictive value (%)
Only HRS(+)	71.7
Only TLR ≥ 1.3(+)	79.1
HRS(−) and TLR ≥ 1.3(−)	23.1
HRS(+) and TLR ≥ 1.3(−)	42.1
HRS(−) and TLR ≥ 1.3(+)	44.4
HRS(+) and TLR ≥ 1.3(+)	88.2

Abbreviations: HRS, high risk stigmata; TLR, tumor‐to‐liver ratio of the SUV.

### 3.2 Effects of TLR in the HGD + IPMC group

The median TLR values for HGD (n = 26) and IPMC (n = 22) patients were 1.4 (0.7‐8.7) and 2.2 (1.0‐6.6), respectively (*P* = .039 on univariate analysis). The AUC of TLR for predicting IPMC was 0.674 with the optimal cut‐off value of 1.6. HRS was also more frequently observed in the IPMC group (65.4% vs 95.5%, *P* = .013).

Postoperative recurrence was found in two of 26 patients (7.7%) with HGD and 13 of 22 patients (59.1%) with IPMC. The sites of recurrence were the remnant pancreas in two patients with HGD, and the lymph node (n = 5), lung (n = 4), remnant pancreas (n = 4), peritoneum (n = 2), liver (n = 2), and others (n = 4) in patients with IPMC (multiple sites for some patients). The median TLR values were not different between IPMC patients with recurrence (3.0, range 1.1‐5.7) and those without recurrence (2.0, range 1.0‐6.6, *P* = .647).

## 4 DISCUSSION

The number of IPMN patients is increasing because of the development of imaging technology and the increase in examination opportunities.^1^, ^2^, ^3^ Therefore, the accurate diagnosis of malignancy is very important to determine the indications for surgery.^14^ The Fukuoka guideline 2017 mainly uses morphological features, such as the malignant index. For example, HRS includes obstructive jaundice in a patient with a cystic lesion of the head of the pancreas, enhanced mural nodule ≥ 5 mm, and MPD size ≥10 mm.^5^ In addition to CT and MRI, current ERCP and EUS enable IPMN lesions to be examined in greater detail.^1^, ^2^, ^3^, ^5^ However, ERCP and EUS are not minimally invasive or easy examinations. IPMN is also reported to be a risk factor for pancreatitis after ERCP.^15^ A more minimally invasive, simple, and highly accurate test is required.

In the Fukuoka guideline 2017, an increased serum CA 19‐9 level is one of the “worrisome features” suggesting malignancy. The sensitivity of both CA19‐9 and CEA for IPMA degeneration is reported to be high, around 90%, but their specificity is not high, around 40% and 6.1%, respectively.^16^ In the present study, both CA19‐9 and CEA were not significantly suggestive of malignancy in univariate analysis, which may be due to the small number of cases in the present study and the low specificity. In addition, recent studies have reported that the neutrophil to lymphocyte ratio (NLR) was a predictor of HGD/invasive IPMC.^17^, ^18^ The CRP to albumin ratio (CAR) was associated with a worse prognosis for various tumors.^19^ However, in the present study, these tumor markers and inflammation‐based markers failed to differentiate benign from malignant IPMNs, though an increased CA19‐9 level tended to be more frequently observed in the HGD/IPMC group (*P* = .066, Table 1).

So far, there have been several reports suggesting that FDG‐PET/CT is useful to diagnose malignancy in IPMN.^10^, ^11^, ^20^, ^21^, ^22^ Some studies reported that, by using a pre‐defined SUVmax cut‐off value of between 2.5 and 3.0, FDG‐PET/CT can differentiate malignant from benign IPMN.^11^, ^21^, ^23^ Another single‐center study performed ROC curve analysis for 29 IPMN patients and determined that an SUVmax of 2.5 was the optimal cut‐off for the differential diagnosis between benign and malignant IPMNs.^24^ On the other hand, it is well‐known that the SUVmax is not an absolute but a relative value and is affected by various factors.^12^ For example, FDG‐PET/CT systems and acquisition protocols were different from one center to another, which may cause the SUVmax value to vary. To obtain a more universal value, the use of a reference tissue such as the liver or the blood pool has become a well‐used practice in PET for the assessment of malignancy of some tumors.^13^, ^25^ The background (non‐tumor) pancreas was considered inappropriate as a reference tissue for several reasons. First, the intestinal tract is present near the pancreas, and the SUV might be affected by its peristalsis. Second, the pancreas is a small‐volume organ, and it is difficult to measure VOI. Third, if pancreatitis is present, the background pancreatic SUV might be high due to inflammation. Accordingly, in the present study, the blood pool and liver were selected as the reference tissues, and the SUVmax, TBR, and TLR were compared to evaluate the performance of FDG‐PET/CT. As shown in Table 1, all three parameters were significantly higher for HGD + IPMC than for IPMA patients. The ROC curve analysis also showed that the three parameters had a similarly high ability to differentiate HGD + IPMC from IPMA, with the AUCs between 0.792 and 0.807. However, when the individual values of these parameters were plotted separately by the three subgroups (Figure 4), the distribution of the SUVmax was not homogeneous and differed widely, compared to those of the TBR and TLR. In addition, as shown in Table 2, the cut‐off levels of the SUVmax for differentiation between benign and malignant IPMNs varied widely from 1.9 to 3.3 among the three subgroups, while that of the TLR was less variable (1.2 or 1.3). These results may suggest that the measured values of SUVmax are dependent on the PET/CT equipment and imaging protocol, and that the effect of differences in these conditions can be decreased by using the TLR. This issue may have been corroborated by a recent French multicenter study,^26^ in which 99 patients with IPMN, including 24 with malignant lesions, were prospectively collected from 12 institutions. The authors reported that the mean and median SUVmax values did not differ between benign and malignant IPMNs, and the cut‐off could not be determined.

Recently, some studies reported the significance of the TLR as a prognostic factor for lung cancer,^27^ hepatocellular carcinoma (HCC),^28^, ^29^ and gastric/esophageal cancer.^30^ In the present study, multivariate analysis showed that HRS and TLR ≥ 1.3 were independent predictors for malignant IPMN. If both were present, the positive predictive value was as high as 88.2%. In addition, although it is difficult to interpret the increased FDG accumulation in HGD cases in comparison with IPMC cases, it was also suggested that the TLR could differentiate IPMC from HGD and predict postoperative outcomes. These results may be of great interest in clinical practice for treating patients with IPMN.

This study had several limitations. First, the numbers of patients and institutions in this study were small. Second, if the patient had hepatitis or liver cirrhosis, the TBR may be more useful than the TLR, because liver disease may affect the liver SUV mean value. Fortunately, in this study, only two of the patients had chronic hepatitis due to HCV. The laboratory data of one patient showed AST, 44 IU/L; ALT, 39 IU/L; T‐BIL, 1.0 mg/dL. The TLR was 0.9, and the pathological diagnosis was IPMA. The data of the other patient showed AST, 106 IU/L; ALT, 99 IU/L; T‐BIL, 0.6 mg/dL. The TLR was 1.2, and the pathological diagnosis was IPMC. The right liver lobe SUV mean values for 86 patients, excluding two patients with chronic hepatitis, ranged from 1.4 to 3.2 with a median value of 2.1, while those for the two patients with chronic hepatitis were equally 1.7. Therefore, the results of the two patients were included for the analysis. Third, the TLR cut‐off value of 1.3 was not validated by using the data of other hospitals and FDG‐PET/CT machines. It is a future task to clarify the relationship between the TLR value and the malignancy of IPMN by analyzing further cases.

## 5 CONCLUSION

The present study showed that the TLR can be a useful predictor for differentiating benign from malignant IPMNs and may be associated with outcomes such as postoperative recurrence. It is suggested that, in contrast to SUVmax, the TLR may reduce the variance due to different PET/CT equipment and imaging protocols and can be widely used in multicenter studies.

## DISCLOSURE

Conflict of Interest: None of the authors have any competing interests to disclose regarding this study.
